# Supplementary material for: Frailty prevalence in older adults with atrial fibrillation: A cross-sectional study in a resource-limited setting
Source: PLoS One. 2024 Oct 24;19(10):e0312498. doi: 10.1371/journal.pone.0312498 (PMC11500909; doi:10.1371/journal.pone.0312498)
Supplement: S1 Table — (DOCX) [file pone.0312498.s001.docx]

| **Model** | | **AIC*** | **Residual deviance** | **Degrees of freedom** | **G^2^**** | **P-value** |
| --- | --- | --- | --- | --- | --- | --- |
| Complete Independence | $log \mu_{ijk}=\lambda+\lambda_{i}^{Cg}+\lambda_{j}^{Fu}+\lambda_{k}^{Fr}$ | 90.95 | 48.659 | 4 | 48.7 | < 0.001 |
| Joint Independence | $log \mu_{ijk}=\lambda+\lambda_{i}^{Cg}+\lambda_{j}^{Fu}+\lambda_{k}^{Fr}+\lambda_{ij}^{\left( Cg \right)\left( Fu \right)}$ | 79.21 | 34.917 | 3 | 34.9 | < 0.001 |
| Joint Independence | $log \mu_{ijk}=\lambda+\lambda_{i}^{Cg}+\lambda_{j}^{Fu}+\lambda_{k}^{Fr}+\lambda_{ik}^{\left( Cg \right)\left( Fr \right)}$ | 78.01 | 33.712 | 3 | 33.7 | < 0.001 |
| Joint Independence | $log \mu_{ijk}=\lambda+\lambda_{i}^{Cg}+\lambda_{j}^{Fu}+\lambda_{k}^{Fr}+\lambda_{jk}^{\left( Fu \right)\left( Fr \right)}$ | 69.94 | 25.643 | 3 | 25.6 | < 0.001 |
| Conditional Independence | $log \mu_{ijk}=\lambda+\lambda_{i}^{Cg}+\lambda_{j}^{Fu}+\lambda_{k}^{Fr}+\lambda_{ij}^{\left( Cg \right)\left( Fu \right)}+\lambda_{ik}^{\left( Cg \right)\left( Fr \right)}$ | 66.27 | 19.97 | 2 | 20.0 | < 0.001 |
| Conditional Independence | $log \mu_{ijk}=\lambda+\lambda_{i}^{Cg}+\lambda_{j}^{Fu}+\lambda_{k}^{Fr}+\lambda_{ij}^{\left( Cg \right)\left( Fu \right)}+\lambda_{jk}^{\left( Fu \right)\left( Fr \right)}$ | 58.20 | 11.901 | 2 | 11.9 | 0.003 |
| Conditional Independence | $log \mu_{ijk}=\lambda+\lambda_{i}^{Cg}+\lambda_{j}^{Fu}+\lambda_{k}^{Fr}+\lambda_{ik}^{\left( Cg \right)\left( Fr \right)}+\lambda_{jk}^{\left( Fu \right)\left( Fr \right)}$ | 56.99 | 10.695 | 2 | 10.7 | 0.005 |
| Homogeneous Association | $log \mu_{ijk}=\lambda+\lambda_{i}^{Cg}+\lambda_{j}^{Fu}+\lambda_{k}^{Fr}+\lambda_{ij}^{\left( Cg \right)\left( Fu \right)}+\lambda_{ik}^{\left( Cg \right)\left( Fr \right)}+\lambda_{jk}^{\left( Fu \right)\left( Fr \right)}$ | **51.81** | **3.5191** | 1 | 3.5 | 0.061 |
| Saturation | $log \mu_{ijk}=\lambda+\lambda_{i}^{Cg}+\lambda_{j}^{Fu}+\lambda_{k}^{Fr}+\lambda_{ij}^{\left( Cg \right)\left( Fu \right)}+\lambda_{ik}^{\left( Cg \right)\left( Fr \right)}+\lambda_{jk}^{\left( Fu \right)\left( Fr \right)}+\lambda_{ijk}^{\left( Cg \right)\left( Fu \right)\left( Fr \right)}$ | - | - | - | 0 | 1 |

**S1 Table. Summary of evaluated log-linear models** for a 3-way contingency table involving the variables frailty, cognitive impairment, and functional dependence. Based on AIC and residual deviance (in bold), the model of homogeneous association was chosen as the most appropriate statistical method for our population. * Akaike information criterion (AIC); ** Likelihood Ratio Chi-Square (based on the relationship between the observed and expected frequencies).
