# Supplementary material for: Frailty prevalence in older adults with atrial fibrillation: A cross-sectional study in a resource-limited setting
Source: PLoS One. 2024 Oct 24;19(10):e0312498. doi: 10.1371/journal.pone.0312498 (PMC11500909; doi:10.1371/journal.pone.0312498)
Supplement: S2 Table — Based on the data (in bold), there is no evidence to support the conclusion that there is a simultaneous interaction between cognitive impairment, frailty, and functional dependence. (DOCX) [file pone.0312498.s002.docx]

| **Coefficients** | **Estimate** | **Standard error** | **Z value** | **Pr(>\|z\|)** |  |
| --- | --- | --- | --- | --- | --- |
| (Intercept) | 3.932 | 0.140 | 28.079 | < 2e-16 | *** |
| Pfeiffer | -1.852 | 0.380 | -4.871 | 1.11E-06 | *** |
| Functional dependence | 0.195 | 0.189 | 1.033 | 0.30153 |  |
| Frailty:Frailty | -2.322 | 0.469 | -4.956 | 7.20E-07 | *** |
| Cognitive impairment:Functional dependence | 0.816 | 0.454 | 1.798 | 0.07223 . |  |
| Cognitive impairment:Frailty | -22.060 | 42247.166 | -0.001 | 0.99958 |  |
| Functional dependence:Frailty | 1.373 | 0.527 | 2.607 | 0.00912 | ** |
| Cognitive impairment:Functional dependence:Frailty | 23.250 | 42247.166 | 0.001 | **0.99956** |  |

**S2 Table. Saturation Model.** Based on the data (in bold), there is no evidence to support the conclusion that there is a simultaneous interaction between cognitive impairment, frailty, and functional dependence. Signification codes: 0 ‘***’ 0.001 ‘**’ 0.01 ‘*’ 0.05 ‘.’ 0.1 ‘ ’ 1
