# Supplementary material for: Frailty prevalence in older adults with atrial fibrillation: A cross-sectional study in a resource-limited setting
Source: PLoS One. 2024 Oct 24;19(10):e0312498. doi: 10.1371/journal.pone.0312498 (PMC11500909; doi:10.1371/journal.pone.0312498)
Supplement: S3 Table — Based on the table, there is no statistically significant relation between geriatric syndromes and oral anticoagulation, except for polypharmacy. (DOCX) [file pone.0312498.s003.docx]

|  | **Oral anticoagulation**  **(n = 181)** | **Not receiving**  **(n = 19)** | ***p*** |
| --- | --- | --- | --- |
|  | **n (%) or**  **m ⇹ IQR** | **n (%) or**  **m ⇹ IQR** |  |
| Number of medications  < 5 medications  > 5 medications  Falls  Cognitive Impairment (*Pfeiffer*)  0 - 2 points  3 - 10 points  Functionality (*Barthel*)  100 points  90-95 points  < 90 points  Frailty (*FRAIL*)  Robust  Prefrail  Frail | 5 ⇹ 3  87 (48.1%)  94 (51.9%)  70 (38.7%)  1 ⇹ 3  128 (70.7%)  53 (29.3%)  90 ⇹ 15  58 (32%)  64 (35.4%)  59 (32.6%)  2 ⇹ 2  39 (21.6%)  83 (45.9%)  59 (32.6%) | 3 ⇹ 2  17 (89.4%)  2 (10.5%)  5 (26.3%)  1 ⇹ 3  14 (73.6%)  5 (26.3%)  95 ⇹ 15  6 (31.5%)  6 (31.5%)  7 (36.8%)  1 ⇹ 2  6 (31.5%)  10 (52.6%)  3 (15.7%) | 0.001396  0.4182  0.9958  0.9207  0.4292 |

**S3 Table. Oral anticoagulation prescribed according to Geriatic syndromes.** Based on the table, there is no statistically significant relation between geriatric syndromes and oral anticoagulation, except for polypharmacy. n = number of participants; % percentage; m: Median; IQR: interquartile range
