# Supplementary material for: Frailty prevalence in older adults with atrial fibrillation: A cross-sectional study in a resource-limited setting
Source: PLoS One. 2024 Oct 24;19(10):e0312498. doi: 10.1371/journal.pone.0312498 (PMC11500909; doi:10.1371/journal.pone.0312498)
Supplement: S1 Fig — (A) FRAIL questionnaire, (B) Pfeiffer Short Portable Mental Status Questionnaire, (C) Barthel Index. (DOCX) [file pone.0312498.s004.docx]

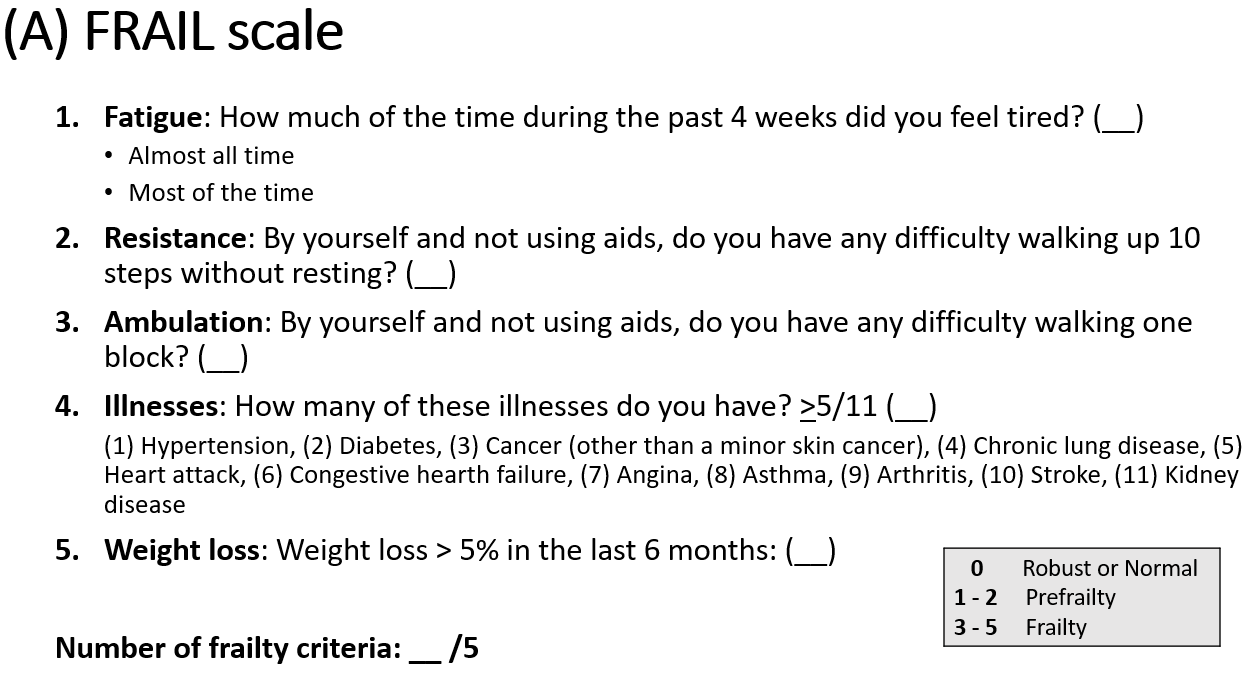

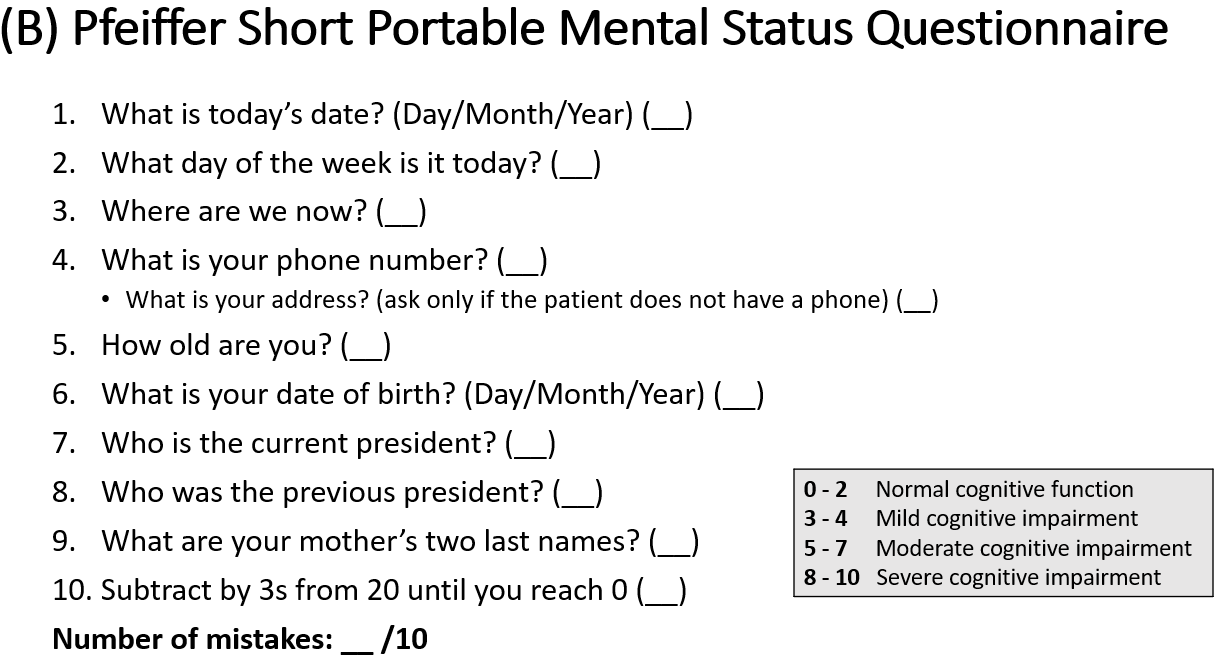


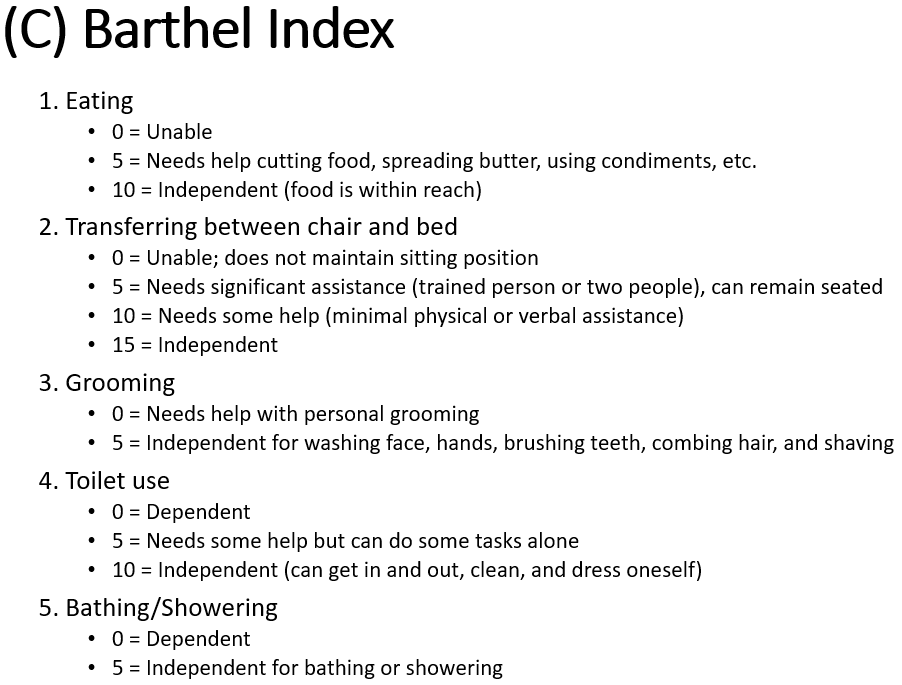

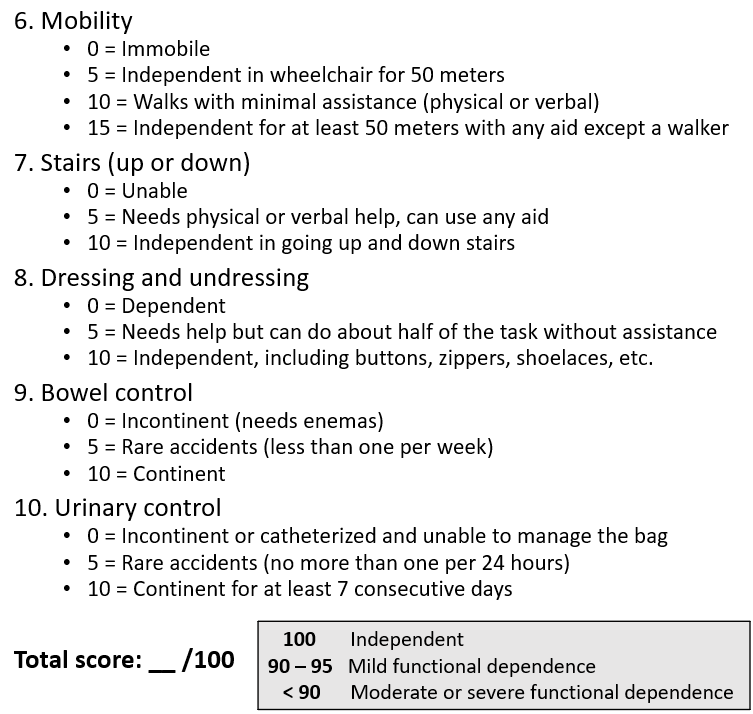


**S1 Figure. Questionnaires used in this study**, translated to english by the authors. (A) FRAIL questionnaire, (B) Pfeiffer Short Portable Mental Status Questionnaire, (C) Barthel Index
