# Supplementary material for: Frailty prevalence in older adults with atrial fibrillation: A cross-sectional study in a resource-limited setting
Source: PLoS One. 2024 Oct 24;19(10):e0312498. doi: 10.1371/journal.pone.0312498 (PMC11500909; doi:10.1371/journal.pone.0312498)
Supplement: Attachment — Submitted filename: Rebuttal Letter - Frailty and Atrial Fibrillation.docx [file pone.0312498.s005.docx]

Editorial Board

Public Library of Science Journal

Paul-Adrian Calburean

Academic Editor, Public Library of Science Journal

Maha Ahmed

Afendoulis Dimitrios

Pair reviewers

September 19, 2024

Dear Editorial Team,

We are pleased to submit this rebuttal letter in response to the corrections and suggestions provided for our original research article titled "Frailty prevalence in older adults with atrial fibrillation: a cross-sectional study in a resource-limited setting" (PONE-D-24-28720). The modifications made are as follows:

1. In the abstract, we added information about the influence of frailty in older adults and simplified the description of the questionnaires used in this study.
2. In the study design section, we addressed some of the study's limitations, including inadequate data registration in the hospital and the limited resources and personnel available for data collection.
3. As per your suggestion, we have included an image that visually represents the prevalence of frailty, prefrailty, and robust participants. We believe this visual representation enhances the clarity and impact of the findings in this resource-limited setting.
4. In the Supporting Information, we have included images of the translated versions of the questionnaires used in this study: the FRAIL scale, the Pfeiffer Short Portable Mental Status Questionnaire, and the Barthel Index.

Modifications are highlighted in yellow in the marked-up copy of our manuscript, while corrections to academic writing are highlighted in blue.

Please, address all correspondence concerning this manuscript to me at jose.colchado.v@upch.pe

Thank you for your consideration of this manuscript

Sincerely,

Dr. Jose Guillermo Colchado Vallejos, MD

Research Assistant, Gerontology Institute

Cayetano Heredia Peruvian University
jose.colchado.v@upch.pe

Dr. Gonzalo Daniel Quirós López, MD

Alberto Hurtado “School of Medicine”

Cayetano Heredia Peruvian University

gonzalo.quiros.l@upch.pe

Dr. Tania Tello Rodríguez, MSc

Director, Gerontology Institute

Cayetano Heredia Peruvian University

Geriatrics Department

Cayetano Heredia National Hospital

tania.tello.r@upch.pe

Dr. Henry Alexander Anchante Hernández, MSc

Cardiology Department

Cayetano Heredia National Hospital

ESC Professional Member. ESC ID: 400136

henry.anchante.h@upch.pe
